# Temperature variability and childhood pneumonia: an ecological study

**DOI:** 10.1186/1476-069X-13-51

**Published:** 2014-06-11

**Authors:** Zhiwei Xu, Wenbiao Hu, Shilu Tong

**Affiliations:** 1 School of Public Health and Social Work& Institute of Health and Biomedical Innovation, Queensland University of Technology, Kelvin Grove 4059, Australia

**Keywords:** Children, Pneumonia, Temperature variability

## Abstract

**Background:**

Few data on the relationship between temperature variability and childhood pneumonia are available. This study attempted to fill this knowledge gap.

**Methods:**

A quasi-Poisson generalized linear regression model combined with a distributed lag non-linear model was used to quantify the impacts of diurnal temperature range (DTR) and temperature change between two neighbouring days (TCN) on emergency department visits (EDVs) for childhood pneumonia in Brisbane, from 2001 to 2010, after controlling for possible confounders.

**Results:**

An adverse impact of TCN on EDVs for childhood pneumonia was observed, and the magnitude of this impact increased from the first five years (2001–2005) to the second five years (2006–2010). Children aged 5–14 years, female children and Indigenous children were particularly vulnerable to TCN impact. However, there was no significant association between DTR and EDVs for childhood pneumonia.

**Conclusions:**

As climate change progresses, the days with unstable weather pattern are likely to increase. Parents and caregivers of children should be aware of the high risk of pneumonia posed by big TCN and take precautionary measures to protect children, especially those with a history of respiratory diseases, from climate impacts.

## Background

Pneumonia is the top cause of mortality in children under five years [1]. It is estimated that in 2010, worldwide, there were 120 million episodes of pneumonia in children younger than five [1]. Pneumonia is highly preventable, hence it is particularly important to explore the risk factors which drive the incidence of pneumonia and further to prevent children from being exposed to these risk factors.

Many nutritional, socioeconomic and environmental factors are involved in the occurrence of pneumonia [2-4]. As climate change proceeds, the possible impact of climate factors on pneumonia transmission has attracted increasing research attention [4,5]. Both high and low temperatures have been reported to be associated with increased pneumonia incidence [6,7]. However, the potential impact of temperature variability on childhood pneumonia has not been researched yet, though big temperature changes may influence the function of respiratory system [8].

There are several ways to define temperature variability [9]. For example, the difference in daily maximum and minimum temperatures (i.e., diurnal temperature range (DTR)) [10], and the mean temperature difference from one day to the next (i.e., temperature change between two neighbouring days (TCN)) [11,12]. Previous studies have highlighted that big DTR or TCN may affect the respiratory system of human [10-12], especially for children [13]. We hypothesized that great DTR or TCN might be associated with increase in childhood pneumonia cases, and we used the data on emergency department visits (EDVs) for childhood pneumonia in Brisbane from 2001 to 2010 to test our hypothesis.

## Methods

### Data collection

Data on EDVs from 1^st^ January 2001 to 31^st^ December 2010 classified according to the International Classification of Diseases, 9^th^ version and10^th^ version (ICD 9 and 10) were supplied by Queensland Health. We extracted those cases coded as pneumonia (ICD 9 codes: 480–486; ICD 10 codes: J12–J18) in children aged 0–14 years. Data on climate variables, including maximum and minimum temperatures, rainfall and relative humidity, were obtained from Australian Bureau of Meteorology. DTR was calculated as daily maximum temperature minus daily minimum temperature [10]. Daily mean temperature was the average of daily maximum and minimum temperatures, and TCN was calculated as mean temperature of the current day minus mean temperature of the previous day [11]. Data on air pollutants, including daily average particular matter ≤ 10 μm (PM_10_) (μg/m^3^), daily average nitrogen dioxide (NO_2_) (μg/m^3^) and daily average ozone (O_3_) (ppb), were retrieved from the Queensland Department of Environment and Heritage Protection. Ethical approval was obtained from the Human Research Ethics Committee of Queensland University of Technology (Australia) prior to the data collection (number: 1000001168).

### Data analysis

Distributed lag non-linear model (DLNM) was developed to incorporate both lagged and the non-linear effects of temperature on mortality or morbidity [14,15]. Previous studies have revealed that there might be a lagged effect of temperature variability on human health, and the relationship between temperature variability and respiratory diseases appears to be non-linear [11-13,15]. Thus, we used DLNM to incorporate the non-linear and lagged effect [14]. A quasi-Poisson generalized linear regression combined with DLNM was used to quantify the association between DTR (or TCN) and EDVs for childhood pneumonia.

DTR model:

$$Y_{t}\sim \mathrm{quasiPoisson}\left(\mu_{t}\right)$$

$$\begin{array}{ll} \mathrm{Log}\;\left(\mu_{t}\right)=\alpha & +\mathrm{\beta DT}R_{t,l}+\mathrm{ns}\left(T_{t,l},3\right)+\mathrm{ns}\left(RH_{t},3\right)\\ & +\mathrm{ns}\left(PM_{10t},3\right)+\mathrm{ns}\left(O_{3t},3\right)+\mathrm{ns}\left(NO_{2t},3\right)\\ & +\mathrm{ns}\left(\mathrm{Tim}e_{t},8\right)+\eta_{1}\mathrm{Holiday}+\eta_{2}\mathrm{DO}W_{t} \end{array}$$

TCN model:

$$Y_{t}\sim \mathrm{quasiPoisson}\left(\mu_{t}\right)$$

$$\begin{array}{ll} \mathrm{Log}\;\left(\mu_{t}\right)=\alpha & +\mathrm{\beta TC}N_{t,l}+\mathrm{ns}\left(T_{t,l},3\right)+\mathrm{ns}\left(RH_{t},3\right)\\ & +\mathrm{ns}\left(PM_{10t},3\right)+\mathrm{ns}\left(O_{3t},3\right)+\mathrm{ns}\left(NO_{2t},3\right)\\ & +\mathrm{ns}\left(\mathrm{Tim}e_{t},8\right)+\eta_{1}\mathrm{Holiday}+\eta_{2}\mathrm{DO}W_{t} \end{array}$$

Where *t* is the day of the observation; *Y*_*t*_ is the observed daily childhood pneumonia on day *t*; *α* is the model intercept; DTR _*t,l*_ is a matrix obtained by applying DLNM to DTR, TCN _*t,l*_ is a matrix obtained by applying DLNM to TCN, *T*_*t,l*_ is a matrix obtained by applying the DLNM to temperature; *β* is vector of coefficients for *T*_*t,l*_, and *l* is the lag days; *ns(RH*_*t*_*, 3)* is a natural cubic spline with three degrees of freedom for relative humidity; *ns(PM*_*10t*_*, 3)* is a natural cubic spline with three degrees of freedom for PM_10_; *ns(O*_*3t*_*, 3)* is a natural cubic spline with three degrees of freedom for O_3_; *ns(NO*_*2t*_*, 3)* is a natural cubic spline with three degrees of freedom for NO_2_; *ns(Time*_*t*_*,8)* is a natural cubic spline with eight degrees of freedom per year for long-term trend and seasonality; Holiday is the public holiday, and *DOW*_*t*_ is the categorical day of the week with a reference day of Sunday.

Specifically, DTR (or TCN) and lag were incorporated using a “natural cubic spline–natural cubic spline” approach. The model included lags up to 21 days for DTR (or TCN) and mean temperature [16]. We used lags up to 10 days, for all other confounders (i.e., relative humidity, PM_10_, O_3_, and NO_2_).

All data analysis was conducted using the R statistical environment (v 2.15), and “dlnm” package was used to fit the regression. In the sensitivity analysis, we changed the *df* for DTR, TCN and time. We also excluded 2009 data as there was a big pneumonia spike in 2009.

## Results

**Figure 1:**
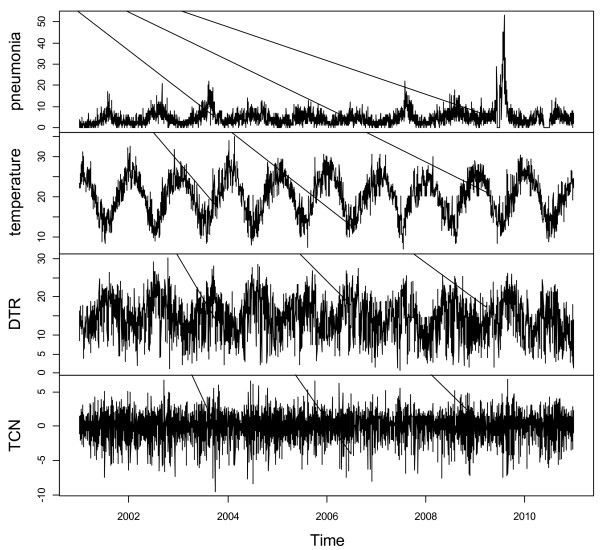
The daily distributions of EDVs for pediatric pneumonia, mean temperature, DTR and TCN in Brisbane, from 2001 to 2010.

**Figure 2:**
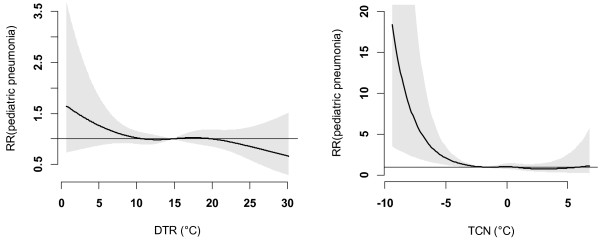
The overall effects of DTR and TCN on pediatric pneumonia in Brisbane, from 2001 to 2010.

**Figure 3:**
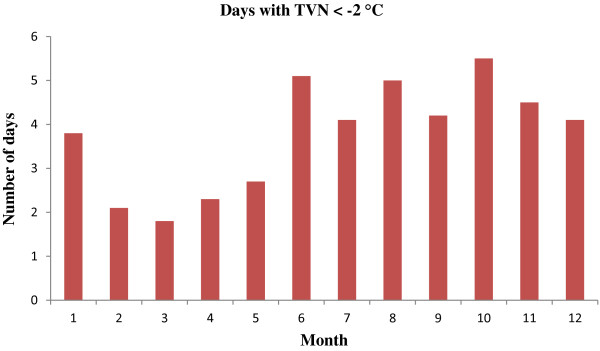
Monthly average number of days with TCN < -2°C.

**Figure 4:**
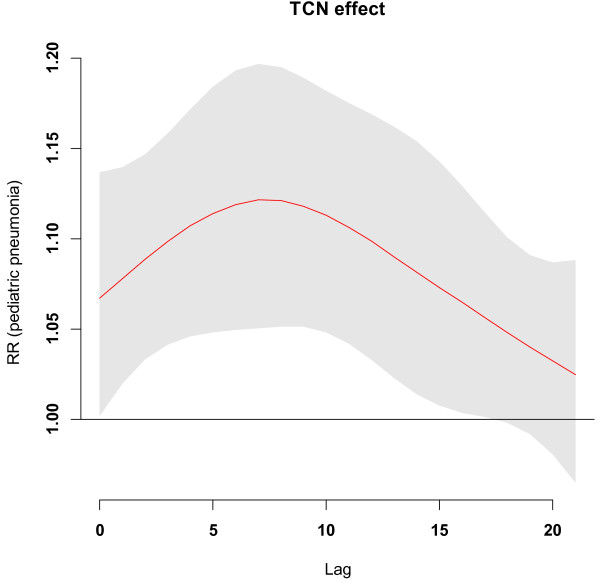
The lagged effect of TCN on childhood pneumonia in Brisbane, from 2001 to 2010.

**Figure 5:**
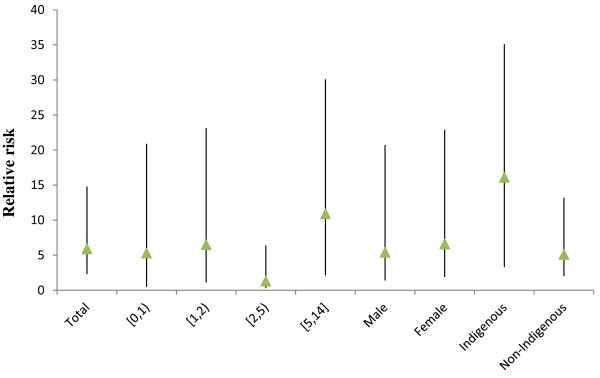
The effect of TCN on the total-, age-, gender- and ethnic-specific childhood pneumonia in Brisbane, from 2001 to 2010.

Figure 1 shows the time-series distributions of childhood pneumonia, mean temperature, DTR and TCN, revealing that there was a seasonal trend in childhood pneumonia, mean temperature, and DTR. The 2009 pneumonia peak (due to H1N1 flu pandemic) is also revealed in this figure. To explore the crude relationship between each variable, we calculated the Spearman correlations between climate variables, air pollutants and childhood pneumonia (Table 1). There was a negative correlation between DTR and mean temperature (r = -0.44, *P* < 0.01). TCN was positively correlated with mean temperature (r = 0.14, *P* < 0.01). Further, DTR was positively correlated with childhood pneumonia (r = 0.21, *P* < 0.01), while no significant correlation between TCN and childhood pneumonia was observed. No correlation coefficient was greater than 0.5, meaning that multi-collinearity is unlikely a big issue in the subsequent modelling.Figure 2 shows the exposure-response relationship between temperature variability and childhood pneumonia (the modelling results). No significant association between DTR and childhood pneumonia was observed. In contrast, a big temperature decrease from one day to the next (TCN < -2°C), increased the risk of childhood pneumonia. Since TCN < -2°C is associated with an increase in childhood pneumonia, we subsequently calculated the number of days with TCN < -2°C every year. There were more than 50 days with TCN below -2°C every year, with most days with temperature drop > 2°C occurring in the second half of each year (June to December) (Figure 3). Figure 4 shows the pattern of lagged effects of TCN on childhood pneumonia, revealing that TCN effect lasted for nearly three weeks. Figure 5 depicts that older children (5–14 years vs. <5 years), female children (vs. male), and Indigenous children (vs. non-indigenous) appeared to be more vulnerable to the TCN impact.

**Table 1:** Spearman’s correlation between daily weather conditions, air pollutants and pediatric pneumonia in Brisbane, Australia, from 2001–2010

	**Mean temperature**	**DTR**	**TCN**	**Relative humidity**	**Rainfall**	**PM**_**10**_	**O**_**3**_	**NO**_**2**_	**Pneumonia**
Mean temperature	1.00								
DTR^†^	-0.44*	1.00							
TCN^†^	0.14*	0.03	1.00						
Relative humidity	-0.06*	-0.33*	0.09*	1.00					
Rainfall	0.16*	-0.49*	-0.03	0.38*	1.00				
PM_10_	0.16*	0.31*	0.08*	-0.29*	-0.31*	1.00			
O_3_	0.03	0.20*	0.03	-0.28*	-0.07*	0.31*	1.00		
NO_2_	-0.67*	0.42*	-0.02	0.13*	-0.14*	0.01	-0.10*	1.00	
Pneumonia	-0.37*	0.21*	-0.01	0.03	0.06*	0.07*	0.10*	0.28*	1.00

*P < 0.01; ^†^DTR, Diurnal temperature range; TCN, temperature change between two neighbouring days.

**Figure 6:**
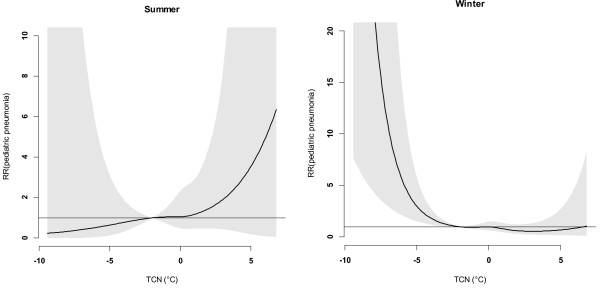
The effect of TCN on childhood pneumonia in summer and winter, from 2001 to 2010.

As there was a distinct seasonality in childhood pneumonia, with the peak in winter, we specifically analysed the TCN impact on childhood pneumonia in summer (December, January, and February) and winter (June, July and August), and found that this impact mainly occurred in winter (Figure 6). However, in summer an increased relative risk of childhood pneumonia was also detected as TCN >0°C, but it was not statistically significant.

**Figure 7:**
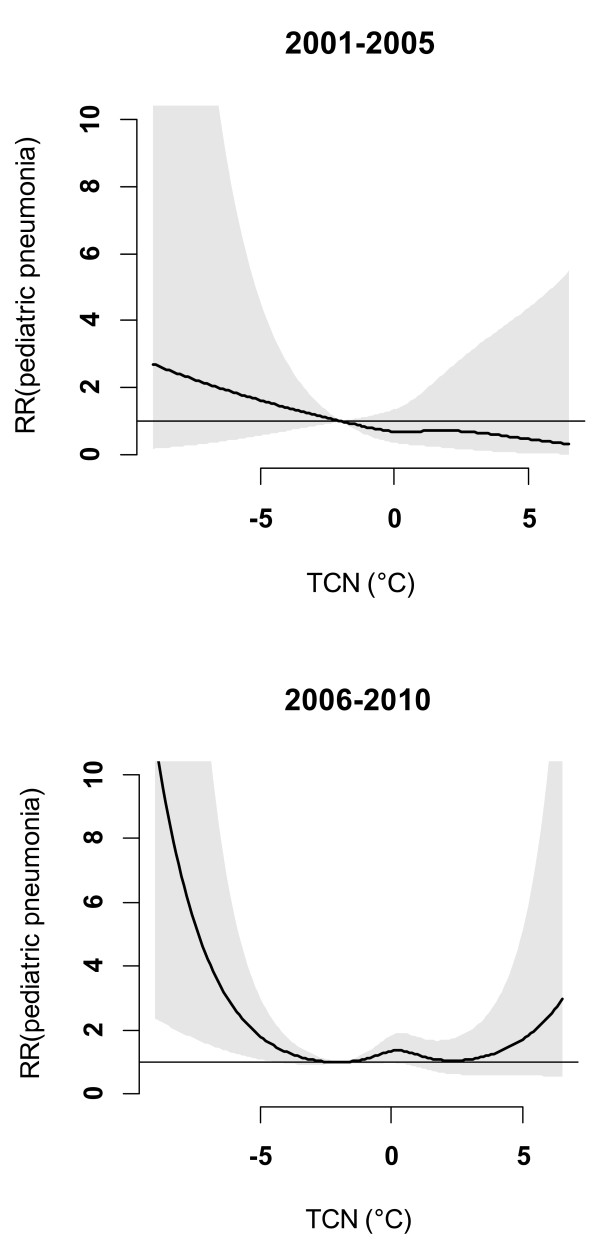
The overall effects of TCN on childhood pneumonia during two periods.

To test whether there was a change over time in the effect of TCN on childhood pneumonia, we splitted the ten years into two periods (2001–2005 and 2006–2010). Figure 7 reveals that the effect of TCN on childhood pneumonia during the 2^nd^ period was much greater than it was during the 1^st^ period.

**Figure 8:**
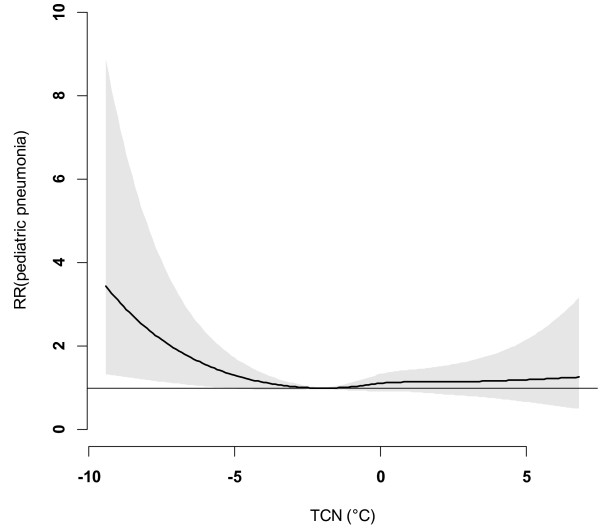
The overall effect of TCN on childhood pneumonia in Brisbane, from 2001 to 2010 (excluding 2009).

**Figure 9:**
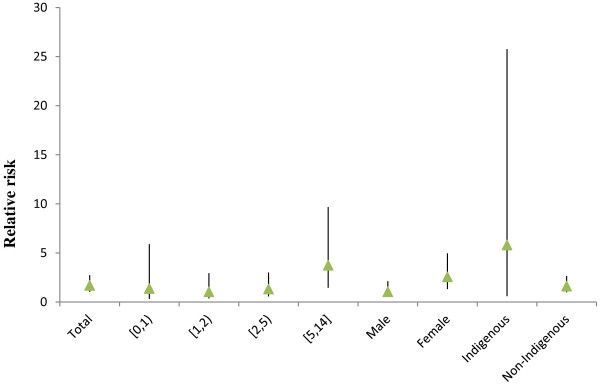
The effect of TCN on the total-, age-, gender- and ethnic-specific childhood pneumonia in Brisbane, from 2001 to 2010 (excluding 2009).

**Figure 10:**
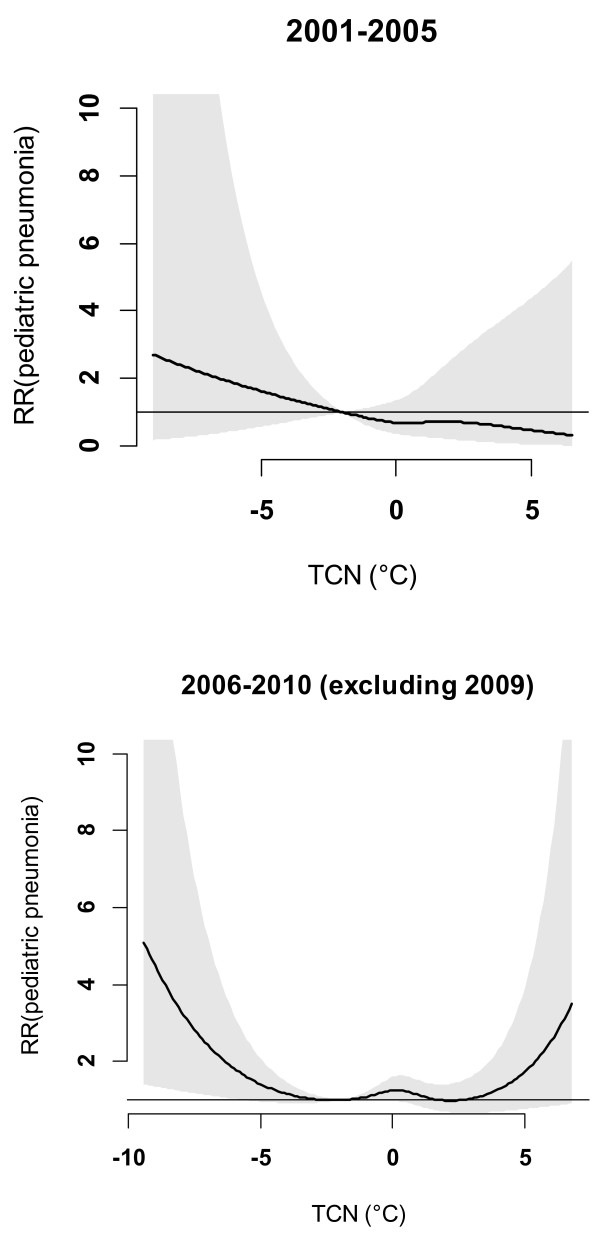
The overall effects of TCN on childhood pneumonia during two periods (excluding 2009).

Figure 8 shows that after excluding the 2009 data, the magnitude of TCN effect on childhood pneumonia in Brisbane reduced, although the shape of the TCN-pneumonia relationship was similar. Figure 9 shows the effects of TCN on childhood pneumonia in different subgroups after excluding the 2009 data, revealing that subgroups vulnerable to TCN effect remained largely unchanged. We also compared the effect of TCN on childhood pneumonia between 2001–2005 and 2006–2010 (without 2009), and found that the TCN effect on childhood pneumonia during the 2^nd^ period was still greater than it was during the 1^st^ period (Figure 10).

## Discussion

This study quantified the impacts of both DTR and TCN on childhood pneumonia and yielded several notable findings. A big temperature decrease from one day to the next (TCN < -2°C) may increase the EDVs for childhood pneumonia, and this effect lasted for around three weeks. Every year, there were more than 50 days with big TCNs, and these big TCNs mainly occurred in winter. Children aged 5–14 years, female children and Indigenous children were particularly at risk. Further, there was a change in the effect of TCN on childhood pneumonia over time. No significant relationship between DTR and childhood pneumonia was observed.

Children are particularly vulnerable to both extreme temperatures [17] and temperature variation [13], due partially to their relatively less-developed thermoregulation capability [18]. In this study, we found that a sharp temperature drop was followed by significantly increased EDVs for childhood pneumonia, and the TCN impact lasted for roughly three weeks. The time lag of TCN impact is probably due to two factors: (1) the temperature change which exceeds certain limits may take a few days to trigger subsequent symptoms in children with underlying conditions; (2) there may be another delay between the onset of symptoms and seeking for medical attention.

Some studies have observed significantly increased respiratory-related mortality associated with big TCN [12] while others did not find significant effects [11]. Our study stands out of previous studies by specifically focusing on childhood pneumonia and controlling for a range of possible confounders. In this study, we also found age, gender and Indigenous-status modified the relationship between TCN and pneumonia. The school-aged children (5–14 years) were more sensitive to TCN compared with younger, which might be because they play outdoors more often and thus exposed more to the outdoor temperature change. The difference in vulnerability to TCN between two genders may be due to their body composition [19], though some researchers argued that such an effect is variable among locations and populations [20]. Our results also suggest that Indigenous children were more sensitive to TCN effect compared with non-Indigenous children. Previous studies have reported that the burden of pneumonia in Indigenous children is 10 to 20 fold higher than non-Indigenous children, and they have longer hospital admissions and are more likely to have multiple admissions with pneumonia [21]. Most Indigenous children have limited access to infrastructures, and experience more poverty than non-Indigenous children, possibly resulting in their greater vulnerability to TCN impact [22].

As climate change progresses, not only the global surface average temperature, but also the frequency of unstable weather patterns (e.g., sharp increase/decrease in temperature) will increase [23], which poses a significant challenge to public health sectors. We found that the effect of TCN on childhood pneumonia during 2006–2010 was greater than it was during 2001–2005. This finding indicates that children might be vulnerable to sharp temperature decrease in the future if unstable weather patterns occur as projected. Elucidating the impact of temperature variability on children’s health is essential for the improvement of public health. The findings of our study not only remind parents and children’s caregivers to take good care of children in the days of big TCN, but also imply that government should take temperature variability into account while developing early warning systems for controlling and preventing childhood pneumonia.

This study has two major strengths. First, this is, to our knowledge, the first study to look at the impact of DTR and TCN on childhood pneumonia. Second, the change over time in the TCN effect on childhood pneumonia which we observed in this study may encourage future studies to explore the temporal variability of TCN impacts on children’s health. Two weaknesses should be acknowledged. First, this is a one city study, which means further the interpretation of our findings should be cautious. Second, to some extent, biases in exposure and/or outcome measures may be inevitable because we used the aggregated data on temperature and EDVs for childhood pneumonia.

## Conclusions

No relationship between DTR and childhood pneumonia was observed. A sharp temperature decrease from one day to the next had an adverse impact on childhood pneumonia, especially in winter, and the magnitude of this impact increased in recent years. In addition, that effect of TCN on the risk of contracting pneumonia appeared to differ by age, gender, and race/ethnicity. As climate change continues, unstable weather patterns may be more frequent, and the findings of this study have important implications for public health policy to protect children from the impact of pneumonia.

## Abbreviations

DLNM: Distributed lag non-linear model; DOW: Day of week; DTR: Diurnal temperature range; EDV: Emergency department visit; ICD9: International classification of disease, ninth revision; ICD10: International classification of disease, tenth revision; NO_2_: Nitrogen dioxide; SD: Standard deviation; PM_10_: Particulate matter ≤10 μm; TCN: Temperature change between two neighbouring days.

## Competing interests

The authors declare that they have no competing financial or nonfinancial interests.

## Authors’ contributions

ZX and ST contributed to the design. ZX led in writing the paper. WH and ST contributed to manuscript revision. All authors read and approved the final manuscript.
